# Prediction of Sensory Parameters of Cured Ham: A Study of the Viability of the Use of NIR Spectroscopy and Artificial Neural Networks

**DOI:** 10.3390/s20195624

**Published:** 2020-10-01

**Authors:** Pedro Hernández-Ramos, Ana María Vivar-Quintana, Isabel Revilla, María Inmaculada González-Martín, Miriam Hernández-Jiménez, Iván Martínez-Martín

**Affiliations:** 1 Graphic Expression in Engineering, University of Salamanca, Escuela Politécnica Superior de Zamora, Avenida Requejo 33, 49022 Zamora, Spain; pedrohde@usal.es; 2 Food Technology, University of Salamanca, Escuela Politécnica Superior de Zamora, Avenida Requejo 33, 49022 Zamora, Spain; irevilla@usal.es (I.R.); miriamhj@usal.es (M.H.-J.); ivanm@usal.es (I.M.-M.); 3 Analytical Chemistry, Nutrition and Bromatology, University of Salamanca, Calle Plaza de los Caidos s/n, 37008 Salamanca, Spain; inmaglez@usal.es

**Keywords:** cured ham quality, artificial neural network (ANN), near-infrared spectroscopy (NIR), sensory analysis

## Abstract

Dry-cured ham is a high-quality product owing to its organoleptic characteristics. Sensory analysis is an essential part of assessing its quality. However, sensory assessment is a laborious process which implies the availability of a trained tasting panel. The aim of this study was the prediction of dry-ham sensory characteristics by means of an instrumental technique. To do so, an artificial neural network (ANN) model for the prediction of sensory parameters of dry-cured hams based on NIR spectral information was developed and optimized. The NIR spectra were obtained with a fiber-optic probe applied directly to the ham sample. In order to achieve this objective, the neural network was designed using 28 sensory parameters analyzed by a trained panel for sensory profile analysis as output data. A total of 91 samples of dry-cured ham matured for 24 months were analyzed. The hams corresponded to two different breeds (Iberian and Iberian x Duroc) and two different feeding systems (feeding outdoors with acorns or feeding with concentrates). The training algorithm and ANN architecture (the number of neurons in the hidden layer) used for the training were optimized. The parameters of ANN architecture analyzed have been shown to have an effect on the prediction capacity of the network. The Levenberg–Marquardt training algorithm has been shown to be the most suitable for the application of an ANN to sensory parameters

## 1. Introduction

Spanish Iberian dry-cured ham is defined by Spanish regulations in Royal Decree 4/2014 (BOE, 2014) as a product produced from the rear limbs of adult pigs with leg and bone subject to the corresponding process of salting and curing-maturation. This product is designated according to its type of genetic purity as “100% Iberian ham” in the case of products from animals with 100% genetic purity of the Iberian breed, and “Iberian ham” in the case of products from animals with at least 50% of their genetic makeup corresponding to the Iberian breed of pig as a result of crossing Iberian mothers with Duroc fathers. As far as the food of the pigs is concerned, hams may be denominated: “acorn-fed” for products from animals slaughtered immediately after feeding exclusively on acorns, grass, and other natural resources of the dehesa pastureland (this feeding system is known as “montanera”), or “fodder-fed” in the case of animals fed on fodder consisting essentially of cereals and leguminous plants, which is handled in intensive exploitation systems.

The organoleptic and nutritional characteristics of Spanish Iberian ham make it a high-quality product which is considered a delicatessen food. There is extensive literature describing the sensory properties of dry-cured ham [1,2,3,4] and more recently even the emotions evoked during the consumption of dry-cured ham have been identified [5]. In order to carry out the sensory analysis of a cured ham, the presence of a trained panel is necessary owing to the number and complexity of the parameters that must be assessed. If the panel is to be suitable, it is necessary to select and train the assessors [4] and to validate the panel regularly to ensure that its members produce trustworthy results [6,7,8]. The validation of a panel requires the holding of controlled sampling sessions which allow the calculation of various indexes of repeatability, precision, and deviation by the assessors [4]. In the food industry, the sensory information of a product is essential, but it is very difficult and time-consuming to implement a suitably trained and validated sampling panel [9,10]. A system allowing the measuring of the sensory quality of a food in an objective manner, without the need for a sampling panel, would reduce the variability associated with human perception and the time needed for carrying out this kind of analysis [11].

It would be of great interest to be able to replace sensory evaluation with faster and simpler instrumental analysis. In this way, near-infrared spectroscopy (NIRS) is an objective and non-destructive method that has been used to predict the quality parameters of different foodstuffs and it had been evaluated for being implemented in dry-cured ham manufacture [12]. This method can be very useful for testing bulk material with little or no sample preparation. The chemical profile obtained by NIR can be successfully linked to specific sensory attributes for expanded snacks [13], for the evaluation and classification of cooked ham [14], for the prediction of lamb meat tenderness assessed by sensory analysis [15], and for the prediction of some quality defects of dry-cured ham samples [16].

However, as NIRS data requires complex statistical treatment, chemometrics has become an established technique for handling this type of data. Among chemometric methods, artificial neural networks (ANNs) are a well-known mathematical tool which is widely used together with the NIR technique in the case of many problems in meat production and technology such as the quality control of raw material, meat processing, shelf-life evaluation, detecting off-flavors, or authenticity assessment [17]. Artificial intelligence methods were mainly investigated for the assessment of meat sensory qualities such as the tenderness, color, or marbling score/level [18]. In relation to dry-cured ham, ANNs have been used for the classification of hams according to the maturation time [19], the identification of feeding and the ripening time of ham [20], and for the assessment of the curing of hams [21]. To our knowledge, there is no data in the literature on the prediction of sensory attributes in dry-cured ham based on NIR information by ANNs.

Taking into account the economic importance of dry-cured ham, the prediction of its sensory characteristics by means of a rapid instrumental technique is an interesting challenge. The aim of this study was to examine the feasibility of using artificial neural networks for predicting ham sensory parameters. Several algorithms, the number of neurons in the hidden layer, and the initial configuration have been tested in order to ascertain the best ANN architecture for predicting the sensory parameters.

## 2. Materials and Methods

### 2.1. Samples

A total of 91 samples of cured ham were analyzed. All the samples were of the fat-marbled part known as “la maza” which is the largest and tastiest part of the ham. The samples were obtained by cutting with a knife along a line passing through the thickest part of dry-cured hams as described by González-Casado et al. [4]. The production and maturation of the samples was carried out in Guijuelo in the province of Salamanca in the traditional manner over 24 months. The ham samples were selected to represent the greatest possible variability within products denominated Spanish Iberian dry-cured ham. Hams were therefore analyzed from pigs with “100% Iberian” genetics together with hams denominated “Iberian” from animals with at least 50% of their genetic makeup corresponding to the Iberian breed of pig, obtained from crossing Iberian mothers with Duroc fathers The samples analyzed included hams from the montanera, from animals fattened extensively with acorns and pasture for 90 days prior to slaughter, and fodder-fed ham from animals fed on commercial feed and pasture in an extensive system. In keeping with these parameters the distribution of the samples analyzed was as follows: 13 samples from 100% Iberian animals fed in montanera (IM), 53 samples of Iberian animals fed in montanera (CM), and 25 samples of fodder-fed Iberian animals (CC).

Once they have been cut the samples are vacuum-packed until they come to be analyzed. The packages were opened 1 h before being tested by the panel.

### 2.2. Sensory Evaluation

Ten assessors (aged from 20 to 50) participated in the study. All of them had previous experience in quantitative descriptive analysis (QDA) of dry-cured hams and were staff at the University of Salamanca. The training was carried out by using products of reference so as to stimulate the generation of terminology during 5 sessions. The assessors did not discuss data, terminology, or samples after each taste session; feedback was provided by the facilitator based on the statistical analysis of the taste session data [22]. The attributes selected for visual appearance, flavor, and texture description of the sample are shown in Table 1. A structured scoring scale was used in which 0 indicated the absence and 9 the high intensity of the attribute. The accuracy of the panel was assessed by studying its reproducibility and repeatability as a whole according to the methodology described by Pérez-Elortondo et al. [23]. To do this, the same ham is analyzed twice in the same session and again in a later session.

Four samples per session were analyzed and a total of 25 sessions were held. Samples were coded with three-digit random numbers and individually presented to the assessor. The average score of the ten assessors for each sample was recorded and used in the statistical analysis. In order to analyze the data, a two-way ANOVA and a post-hoc test (Tukey) were carried out to check for significant differences between samples. In order to investigate the relationship between the two attributes, the Pearson correlation coefficients have been calculated.

### 2.3. NIR-Chemometric Methods

The near-infrared spectra of the samples were obtained using a Foss NIRSystem 500 (Hillerod, Denmark). This equipment was coupled with a fiber-optic probe (1.5 m 210/210, Ref. No. R6539-A) and a 5 cm × 5 cm window quartz. The window was applied to the surface of ham directly without any preparation. The Foss NIRSystem 500 is equipped with four reflectance detectors (PBs elements) that, in order to minimize the specular reflectance, are placed at a 45° angle to the sample surface. The reference of the probe is a ceramic plate. The spectra of the sample were recorded in the 1100–2000 nm range at intervals of 2 nm, which means that a total of 451 pieces of reflectance data were obtained for each sample. The spectra above 2000 nm were not recorded as the OH groups that may be present in the optical fiber produce a significant attenuation of the signal. For each recording, 32 scans were performed for both the reference and sample. Indeed, all the samples were analyzed in triplicate to minimize sampling errors. The spectra of each sample were averaged and the logarithm of the reciprocal of the reflectance values was calculated to transform it into absorbance (A = log 1/R).

### 2.4. Artificial Neural Network

The multilayer perceptron (MLP) feedforward artificial neural network (ANN) was used for processing the absorbance values obtained. The 451 values of NIR absorbance feed the input layer which had 451 neurons. The hidden layer has a variable number of neurons between 1 and 30 depending on the sensory parameter predicted and used the hyperbolic tangent sigmoid function. The output layer used the pure linear transfer function and has only one neuron. This neuron shows the estimated value of one of the sensory parameters. The use of a known seed value number to randomly initialize the weight and bias matrix allows us the reproducibility of data [24]. The pairs of NIRS-sensory data, i.e., input-output data, were randomly divided for all ANNs into three sets as follows: training, validation, and test set that accounts for the 70%, 15%, and 15% of the data respectively. Then, 28 ANN architectures, one for each of the sensory parameters, were optimized. Tests were carried out with the Scaled Conjugate Gradient Backpropagation and Levenberg–Marquardt Backpropagation training algorithms. The Deep Learning Toolbox of MatLab (MathWorks^®^) in its R2018 version was the software used for all the tests.

## 3. Results

### 3.1. Sensory Analysis

Sensory analysis is referred to as the evaluating of perceptible characteristics or organoleptic properties of ham termed as “attributes”. The technique used for the sensory characterization of ham was the quantitative descriptive analysis (QDA), which is the technique most frequently used with training panels to describe the sensory properties of different types of dry-cured ham [25,26,27]. In the case of dry-cured ham, the sensory attributes are the result of the interaction between the quality of the fresh material and the biological changes which occur during the processing [28,29], which are influenced by both the technological process and the duration of the maturation [30]. The results obtained in the tasting sessions for each of the attributes described in this study can be seen in Table 2.

As far as the appearance profile is concerned, the attributes of veined, fat color, color intensity, exudate, and white dots present statistically significant differences (*p* < 0.05) which depend on the type of ham analyzed. Hams from IM pigs therefore gave lower scores for the attributes of veineds, fat color, exudate, and white dots. For the first two attributes, the maximum scores corresponded to CC hams while for the exudate and white dots CM hams obtained the highest scores. For all these parameters both the feeding and genetics of the animals had a significant influence on the scores awarded by the samplers. All the samples analyzed in this study are from Iberian pigs, either 100% pure or crossed at 75 or 50%; a characteristic of this breed is a high intramuscular fat (IMF) content owing to both the rearing system and the genetic features of the pig breed [31]. The high content in muscular fat has been related to the veined and exudate parameters [32,33]. In this study, higher values of these parameters were found for crossbred samples (CM and CC). A higher color intensity was found in animals fed in montanera (IM and CM) related to greater exercise and to yellower fat which was probably due to the higher unsaturation of the fat. Color homogeneity is the only visual attribute which presents no significant differences between the groups. This attribute is more closely related to the dry-curing process, which includes the origin of the formation of compounds associated with the characteristic color of dry-cured meat products [34].

In relation to flavor, all parameters have shown statistically significant differences between the three groups of hams analyzed except for the parameters of atypical aroma, sourness, and saltiness. These parameters are associated with defects present in cured hams and appear to be related to the technological processes and maturation conditions of the product. CM hams showed the highest values of odor, flavor intensity, fat flavor intensity, cured flavor, sweetness, and aftertaste, while CC hams showed in general the lowest values of the same parameters with the exception of fat flavor intensity which was lower in IM hams.

The factors of feeding and genetics of the animal have a different influence according to the attributes; only odor, flavor intensity, cured flavor, and aftertaste presented significant differences for both factors. Therefore, feeding had a significant influence on the parameters of cured aroma, pig aroma, sweetness, and aftertaste while genetics had a significant influence regarding rancidity, aroma and flavor, fat flavor, and atypical flavor. Lipolysis and proteolysis are the main biochemical reactions involved in the generation of a wide range of volatile compounds [35,36]. The fat present in both the muscles and the subcutaneous tissue appears to be of great importance in the entire flavor of Iberian hams and means that the flavor of this type of product is highly complex. The volatile compounds which contribute towards the odor and flavor of dry-cured ham are mainly generated during the maturation process from the oxidation of the fatty acids and Maillard reactions [37]. To a lesser extent, volatile compounds are formed from mold and yeast and Iberian ham has a particularly high concentration [38]. There are also a small number of compounds which are directly accumulated in pig fat deposits from feeding [39], which would justify the low number of parameters with significant differences which can be directly attributed either to the different genetic purity or to the feeding system of the pigs.

The results obtained in the texture parameters show that chewiness, gumminess, and heterogeneity are the only attributes which do not present significant differences in the scores awarded by the assessors for the three groups of hams analyzed. IM hams have the lowest scores in the attributes of juiciness and fatness and the highest in the attributes of hardness and chewing residue. High intramuscular fat (IMF) content thus appears to have a very remarkable effect on the texture of dry-cured ham [32], increasing the juiciness and decreasing the hardness and fibrousness [32,39]. In our study, we found a correlation between the veined and texture parameters. The lower the veined value the lower the juiciness and fatness and the higher the hardness. During the processing of dry-cured ham proteolysis, that is affected by an important number of factors such as pH of fresh ham, anatomic location, temperature, water, or salt content [40,41,42], is one of the main biochemical reactions. In fact, proteolysis is considered to be the major contributor to texture changes [43,44].

Previous studies suggest that the color of Spanish dry-cured hams has a strong correlation with their texture [45]. From our results, a negative correlation (at the 0.01 level) can be observed between the attributes of color fat and color homogeneity and the parameters of texture: hardness, chewiness, gumminess, heterogeneity and chewing residue. Likewise, they show a positive correlation with the fatness attribute. For its part, the color intensity attribute presents no correlations with any of the texture parameters analyzed.

### 3.2. Spectral Characteristics 

**Figure 1:**
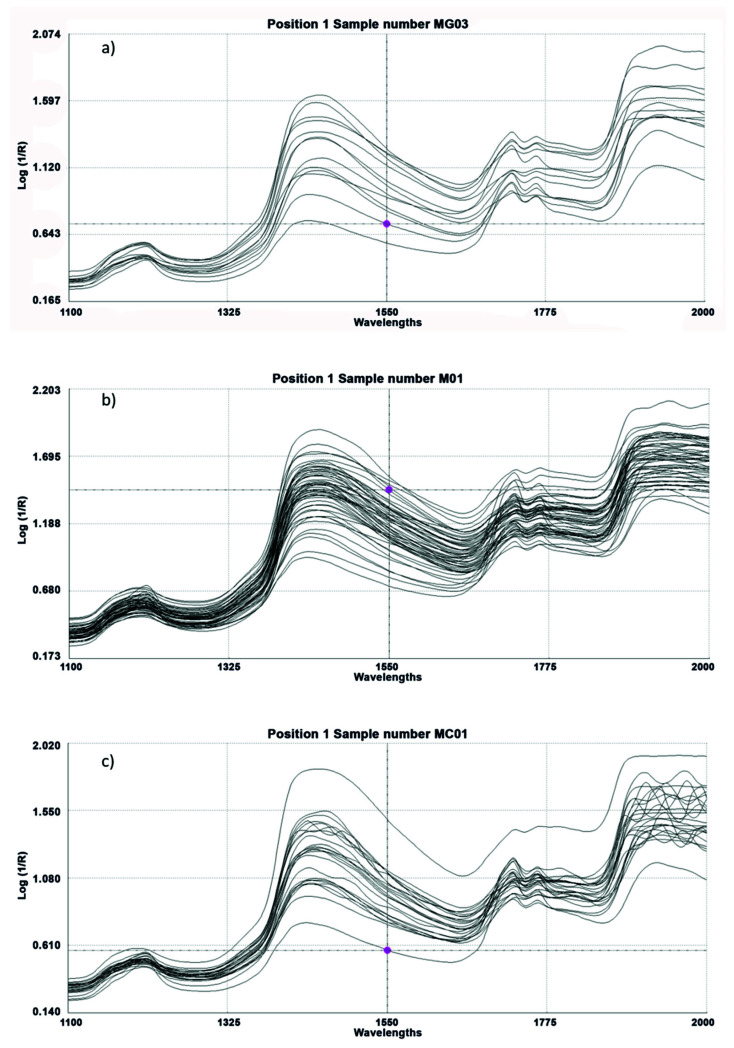
Near Infrared Spectroscopy (NIR) spectra obtained from a remote reflectance fiber-optic probe applied directly to samples of ham. (**a**) 100% Iberian animals fed in “montanera2 (IM), (**b**) Iberian animals fed in “montanera” (CM) and (**c**) fodder-fed Iberian animals (CC).

In the ham samples analyzed by the assessors, the register of their spectra was carried out by using NIRS technology and a remote reflectance fiber-optic probe. The mean spectral curves of the registered samples are shown in Figure 1.

The wavelengths responsible for the NIR are due to C-H stretching combinations with other vibrational modes, in addition to the strong absorptions shown by the molecules containing N-H, S-H, and P-H. In this way, the NIR spectra obtained allow us to establish a relationship between different chemical molecules and functional groups and the sensory parameters analyzed in the ham. Group C-oil is therefore strongly related to the perception of the saltiness and fatness of the ham and group C-Cl is related to saltiness and rancidity. Group C-O-oil is related to the fibrousness of the product and group SH-SH is strongly related to texture parameters such as fibrousness, chewiness, and gumminess. In addition to these chemical groups which provide greater weight and higher correlation coefficients in the NIR predictive models, we have been able to relate other groups with some of the sensory attributes analyzed as is shown in Table 3.

### 3.3. Artificial Neural Network (ANN) 

The results obtained in the sensory analysis of the hams presented in this study allow the availability of sufficiently heterogeneous products, owing to which we have a satisfactory sample for assessing the application of neuronal networks in the prediction of the same. ANNs become useful in those cases in which the rules underlying the data are unknown or only partially known [17].

**Figure 2:**
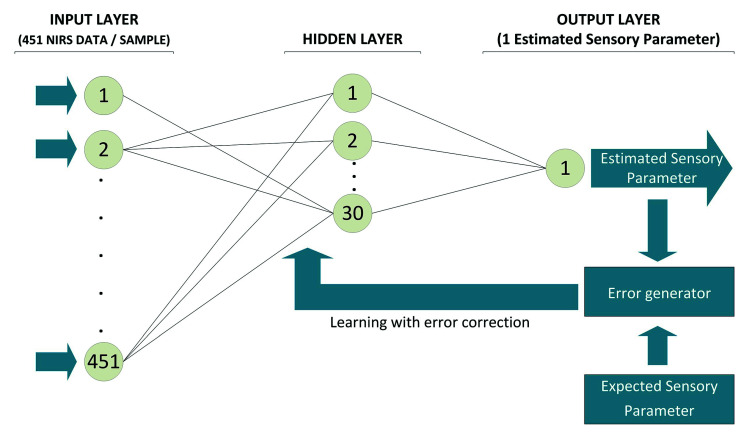
Structure of feedforward multi-layer Artificial Neuronal Network (ANN) for calculating sensory parameters of cured ham; this diagram is repeated for each of the 28 sensory parameters studied.

The neural networks tested were constructed by using the NIR spectra and the sensory parameters such as input and output data respectively. The diagram of the network structure used is shown in Figure 2.

The input data were the values obtained from NIR spectra. A total of 451 reflectance data for each sample, corresponding to the 451 log 1/R values obtained between 1100 and 2000 nm were measured every 2 nm. In turn, each register is the result of the measurement of the NIR spectrum at 32 different points of that sample. As output data, we used the average values of the sensory parameters provided by the assessors for each of the sensory parameters analyzed.

Although the ANN technique has many advantages, model users always spend a great deal of time and effort in the process of parameter training. Identifying the optimum parameters of ANNs can help us avoid an unnecessary waste of time and effort [46]. For this reason, an attempt was made to assess the training algorithm and the network architecture in order to predict dry-cured ham sensory analysis. The ANN training algorithms Scaled Conjugate Gradient (SGC) and Levenberg-Marquardt (LM) were examined. For each of them between 1 and 30 neurons were tested in the hidden layer. In total 1500 networks were analyzed, 30 different values of neurons in the hidden layer with 50 different initial states for each of them. The suitability of the networks obtained was established from the value R^2^ (the R-square between the target and the estimated parameter). For each sensory parameter, the networks with a value exceeding R^2^ > 0.72 in the test set were taken into account, which gives an idea of the number of networks generated with the capacity for predicting this parameter. The results obtained reveal that the parameter with the largest number of networks capable of predicting it were heterogeneity (26.4% of the networks) followed by the parameters of sourness (13.4%), atypical flavor (9.5%), and odor (7.0%). The LM training algorithm allowed the finding of networks for the prediction of all the sensory parameters and was the one providing the largest number of networks with R^2^ > 0.72.

The number of neurons in the hidden layer should be between the input and the output layer size and be determined empirically [47,48]. Networks with from 1 to 30 neurons and three different number of training times (30, 100, and 500) were tested. Table 4 shows the number of neurons providing the best ANN architecture (in terms of obtaining the highest R^2^) for each number of training times assessed. The results show that when the training times increase the number of neurons in the hidden layer necessary for obtaining the network with the highest R^2^ falls, in such a way that in the case of 100 and 500 training times it will not be necessary to test a number of neurons higher than 10 in the hidden layer. The optimum number of neurons in the hidden layer and training times was different for each of the sensory parameters analyzed in the ham. Owing to the variability of the results it was decided to assess the sensitivity of the parameters of ANN architecture. Following the model proposed by [46] to identify the sensitivity of parameters of ANN architecture, the sensitivity index (S) was calculated.

Our results show that there is a direct relationship between the number of neurons in the hidden layer and the number of training times and the R^2^ for all the parameters analyzed with the exception of the “rancidity aroma.” According to our results, 23 of the 28 estimated sensory parameters in dry-cured ham are more sensitive to the number of neurons in the hidden layer than the number of training times in ANN architecture. Only the visual color intensity parameter, the flavor intensity and atypical flavor parameters, and the juiciness and chewing residue texture parameters show more sensitivity to the training times.

**Figure 3:**
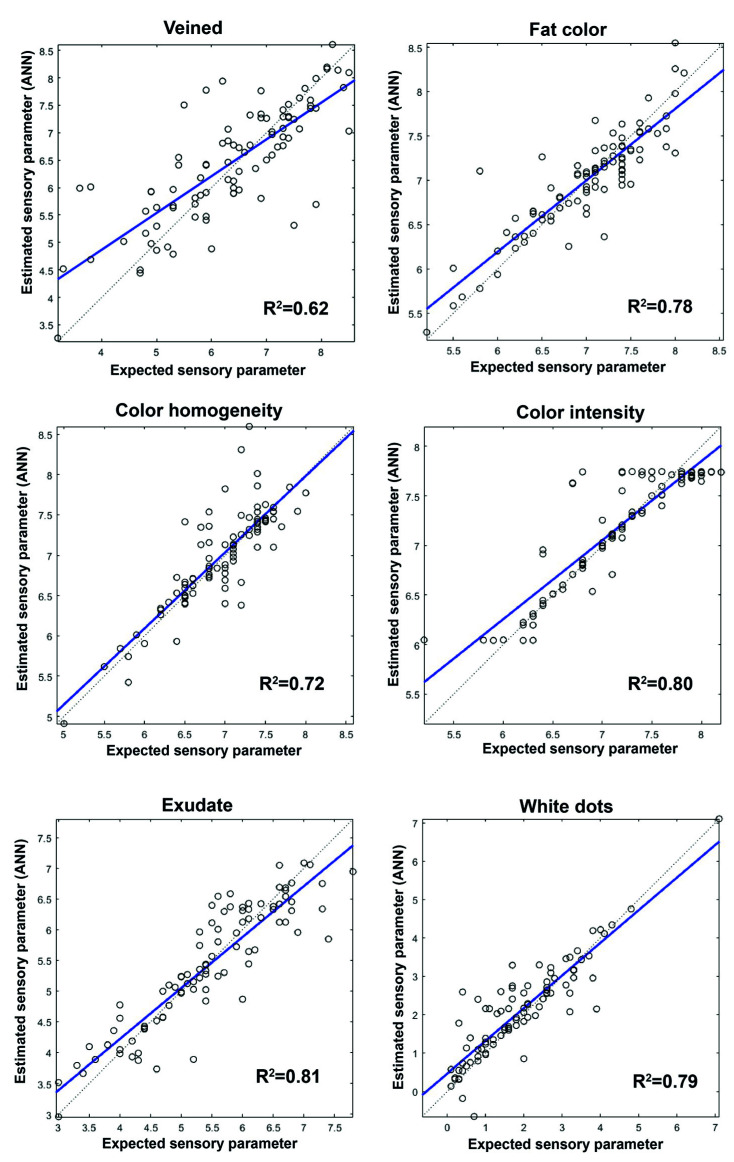
Comparison of the reference values (target) with the values predicted by ANNs for visual parameters and R^2.^

**Figure 4:**
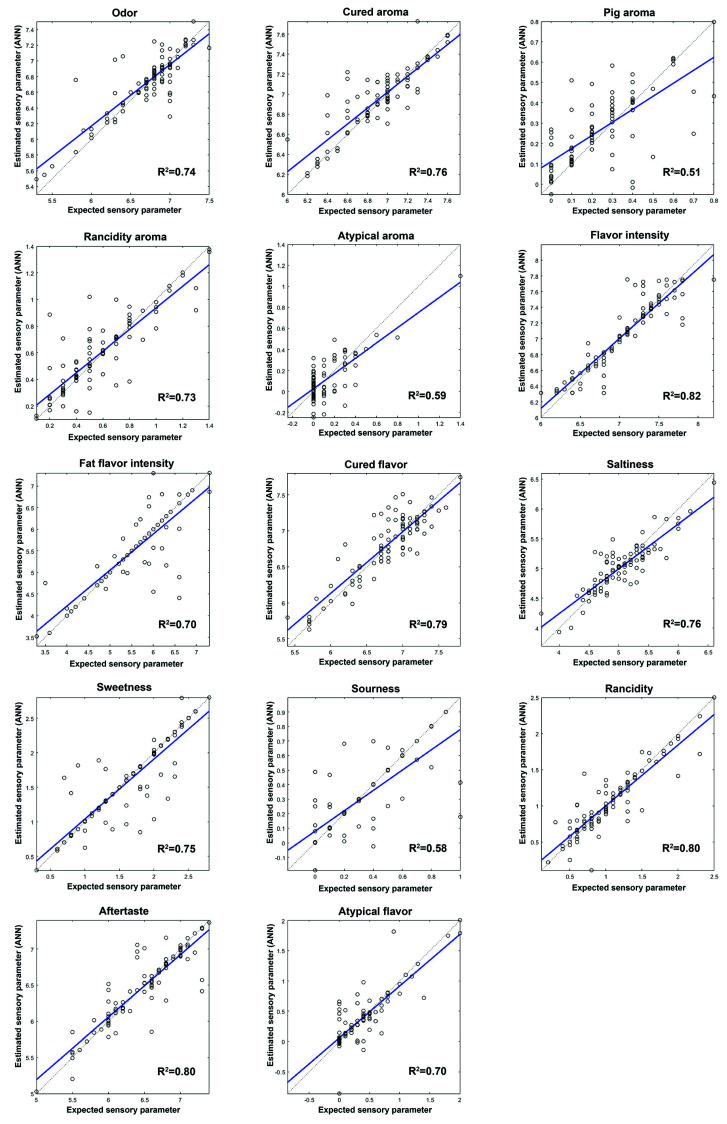
Comparison of the reference values (target) with the values predicted by ANNs for flavor parameters and R^2.^

**Figure 5:**
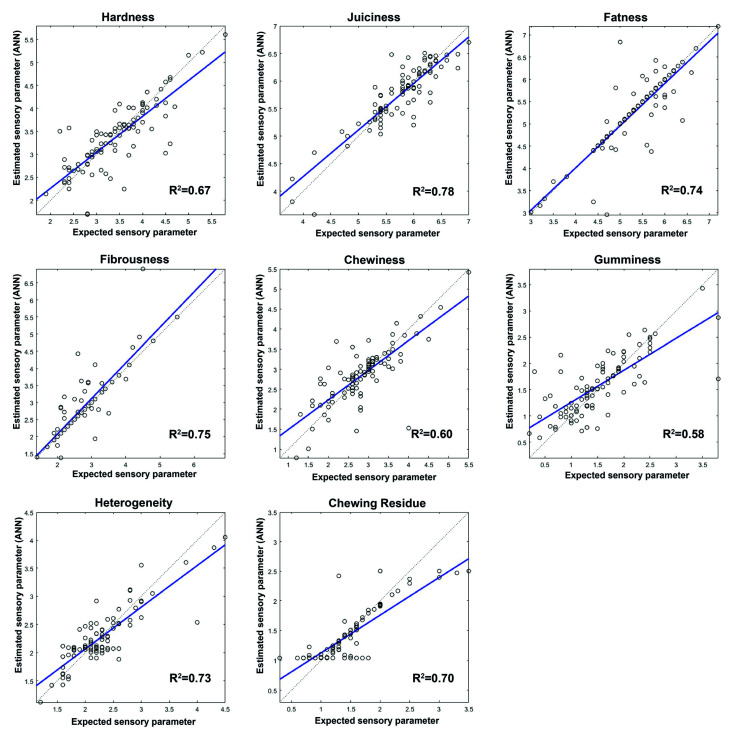
Comparison of the reference values (target) with the values predicted by ANNs for texture parameters and R^2.^

Once the best network architecture had been established, the sensory parameters of dry ham were predicted. Figure 3, Figure 4 and Figure 5 show the prediction graphs and the R^2^ values and the adjustment lines obtained. The R^2^ values obtained vary between 0.51 for the pig aroma parameter to 0.82 for the flavor intensity parameter.

As far as the appearance profiles (Figure 3) are concerned, all of them show a satisfactory correlation between the target and the values predicted by the network. In relation to the odor and flavor of dry-cured hams (Figure 4), the values with the highest correlation were aftertaste, rancidity, and flavor intensity. The results obtained for the texture parameters (Figure 5) show that juiciness, fatness, and fibrousness provide the networks with the best adjustments. Of the 28 sensory parameters analyzed no relation was found between the prediction capacity of the network and the significant differences in the values given by the assessors, whether owing to the purity effect of the breed or to the feeding effect.

The prediction network generated was further tested with 14 samples of ham (a set test) which were neither part of the training nor the validation set. The mean squared errors (MSEs) between the targets and the ANN outputs were assessed (Table 5). The MSEs found varied between 0.0196 and 0.5878; the highest errors occurred in the prediction of the texture and visual parameters. The best results in the prediction of sensory parameters were obtained for the parameters related to flavor. The lower prediction capacity of the network could be seen in the attributes of pig aroma, atypical aroma, sourness, and gumminess with an R^2^ between 0.51 and 0.59 (Figure 4 and Figure 5). However, when the network generated was checked against the set test the number of errors made by the network in the prediction of these parameters is low. The values obtained in all the sensory parameters analyzed suggest that the network generated can be applied satisfactorily to unknown samples.

## 4. Conclusions

The results obtained in this study allow us to conclude that NIR spectral information and the application of ANNs could be an interesting tool for predicting the sensory parameters of dry-cured hams. The sensory analysis of ham requires a great investment of time and a trained panel to carry it out. With the methodology proposed it would be possible to predict the sensory parameters of the ham at the same time as it is sliced. The results showed that these models have the ability to predict the most important sensory parameters for dry-cured ham with relatively high accuracy. Future studies with greater heterogeneity of samples will be necessary to improve the results obtained for some sensory parameters.

## Figures and Tables

**Table 1:** Sensory parameters selected by the assessors and the definition of sensory parameters.

Parameter	Description	Score Criteria	
***Visual***		0	9
Veined	Amount of intramuscular fat	Absence of intramuscular fat	Large amount of intramuscular fat
Fat color	Color shade of the intramuscular fat	Yellow	White
Color homogeneity	Presence or absence of the various shades	Inhomogeneous	Homogeneous
Color intensity	Color intensity of the item	Pink	Red
Exudate	Shine from the separation of fat on the surface	Absence of exudate	High intensity of exudate
White dots	Presence of white dots owing to the precipitation of tyrosine	Absence of white dots	Large number of white dots
***Flavor***			
Odor	Intensity of odor before eating	Low intensity of odor	High intensity of odor
Cured aroma	Odor of cured meat	Low intensity of cured aroma	High intensity of cured aroma
Pig aroma	Odor of abattoir or recently slaughtered pig	Absence of pig aroma	High intensity of pig aroma
Rancidity aroma	Intensity of rancid odor	Low intensity of rancid odor	High intensity of rancid odor
Atypical aroma	Presence of strange odors uncharacteristic of ham	Absence of atypical aroma	High intensity of atypical aroma
Flavor intensity	Sensation of flavors once the product has been placed in the mouth	Low intensity of flavor	High intensity of flavor
Fat flavor intensity	Flavor intensity of the fat fraction	Low intensity of fat flavor	High intensity of fat flavor
Cured flavor	Intensity of cured flavor	Low intensity of cured flavor	High intensity of cured flavor
Saltiness	Intensity of salty taste	Low intensity of saltiness	High intensity of saltiness
Sweetness	Intensity of sweet taste	Low intensity of sweetness	High intensity of sweetness
Sourness	Intensity of acid taste	Low intensity of sourness	High intensity of sourness
Rancidity	Intensity of rancid flavor	Low intensity of rancid flavor	High intensity of rancid flavor
Aftertaste	Persistence of the taste after having eaten the product	Low intensity of aftertaste	High intensity of aftertaste
Atypical flavor	Presence of strange odors uncharacteristic of ham	Absence of atypical flavor	High intensity of atypical flavor
***Texture***			
Hardness	Firmness perception during chewing	Low intensity of firmness	High intensity of firmness
Juiciness	Impression of juiciness during chewing	Low intensity of juiciness	High intensity of rancid odor
Fatness	Appearance of a fatty sensation when chewing the product	Low intensity of fatness	High intensity of fatness
Fibrousness	Perception of fibers during chewing	Low number of fibers	High number of fibers
Chewiness	No. of bites necessary before the item is swallowed	Few bites	Many bites
Gumminess	Tendency to form a ball when the product is chewed	Low intensity of gumminess	High intensity of gumminess
Heterogeneity	Presence or absence of different textures in the item on chewing it	Homogeneity	Lack of homogeneity
Chewing Residue	If remains of the product stay in the mouth once we have swallowed it	Little or no residue	Large amount of residue

**Table 2:** Scores for each sensory attribute of dry-cured ham determined by the sensory panel and results of the statistical analysis of the influence of the feeding and the genetics of the animals.

Sensory Attribute		Mean			*p*-Value	
		IM *	CM **	CC ***	Feeding	Genetics
Visual parameters	Veined	5.37 ^a^	6.20 ^b^	7.22 ^c^	0.000	0.002
	Fat color	6.20 ^a^	7.05 ^b^	7.37 ^c^	0.001	0.000
	Color homogeneity	6.78 ^a^	7.00 ^a^	6.82 ^a^	0.308	0.313
	Color intensity	7.50 ^b^	7.39 ^b^	6.42 ^a^	0.000	0.034
	Exudate	4.42 ^a^	6.00 ^b^	4.78 ^a^	0.000	0.000
	White dots	1.13 ^a^	2.56 ^b^	0.85 ^a^	0.000	0.017
Flavor parameters	Odor	6.34 ^a^	6.88 ^b^	6.50 ^a^	0.019	0.001
	Cured aroma	6.82 ^b^	7.04 ^c^	6.61 ^a^	0.000	0.414
	Pig aroma	0.25 ^a^	0.20 ^a,b^	0.33 ^b^	0.007	0.871
	Rancidity aroma	0.79 ^b^	0.49 ^a^	0.63 ^a,b^	0.235	0.004
	Atypical aroma	0.16 ^a^	0.09 ^a^	0.15 ^a^	0.364	0.414
	Flavor intensity	6.60 ^a^	7.34 ^b^	6.69 ^a^	0.000	0.000
	Fat flavor intensity	4.77 ^a^	5.79 ^c^	5.31 ^b^	0.135	0.000
	Cured flavor	6.47 ^a^	7.00 ^b^	6.35 ^a^	0.000	0.026
	Saltiness	5.03 ^a^	4.98 ^a^	5.16 ^a^	0.139	0.963
	Sweetness	1.49 ^a^	1.83 ^b^	1.24 ^a^	0.000	0.346
	Sourness	0.36 ^a^	0.27 ^a^	0.36 ^a^	0.255	0.381
	Rancidity	1.48 ^b^	0.94 ^a^	1.01 ^a^	0.711	0.000
	Aftertaste	6.23 ^a^	6.73 ^b^	6.02 ^a^	0.000	0.074
	Atypical flavor	0.75 ^b^	0.32 ^a^	0.29 ^a^	0.276	0.001
Texture parameters	Hardness	4.00 ^b^	3.24 ^a^	3.47 ^a^	0.694	0.002
	Juiciness	5.16 ^a^	5.47 ^b^	6.02 ^c^	0.007	0.000
	Fatness	4.78 ^a^	5.50 ^b^	5.15 ^a,b^	0.229	0.004
	Fibrousness	2.93 ^a,b^	2.53 ^a^	3.30 ^b^	0.000	0.474
	Chewiness	2.93 ^a^	2.70 ^a^	3.03 ^a^	0.102	0.581
	Gumminess	1.70 ^a^	1.48 ^a^	1.55 ^a^	0.855	0.366
	Heterogeneity	2.19 ^a^	2.28 ^a^	2.32 ^a^	0.640	0.541
	Chewing Residue	1.80 ^b^	1.24 ^a^	1.61 ^b^	0.068	0.013

* IM: 100% Iberian animals fed in “montanera”; ** CM: Iberian animals fed in “montanera”; *** CC: fodder-fed Iberian animals; ^a,b,c^—Different letters in the same line indicate statistically significant differences (*p* < 0.05) between the three groups of ham samples.

**Table 3:** Sensory attributes for which it has been possible to establish a relationship with the spectral wavelengths and the chemical groups responsible for the perception of this attribute.

Sensory Attribute	Wavelength (nm)	Chemical Structure and Functional Groups
Fat color	1362	CH_3_
	1460	Urea, Starch, Amides
	1536	Amides
	1772	Cellulose
Cured aroma	1510	Protein
	1620	=CH_2_
	1770	Cellulose
	1954	Aromatic ester
Rancidity aroma	1450	Water, Ketone, Starch
	1512	Protein
	1922	Cellulose, Starch
Atypical aroma	1458	Amides
	1482	Amides, Aromatic amides, Cellulose, Urea, Aromatic amines
	1622	=CH_2_
Cured flavor	1416	CH-aromatic compounds
	1512	Protein
	1882	Cellulose
	1982	Amides
Saltiness	1406	H_2_O
	1416	C-Oil *, ROH-H_2_O
	1446	CH_2_, aromatic compounds, starch
	1488	Cellulose, Amines, Aromatic amines
	1520	Urea
	1686	Aromatic compounds
	1866	C-Cl *
	1950	Aromatic amides
Sourness	122	CH_2_
	1538	Starch
Rancidity	1390	CH_2_
	1454	Starch
	1506	-NH, Protein
	1528	Aromatic amines
	1682	C-Cl *
Hardness	1416	OH-H_2_O, Alcohol/aromatic compounds
	1502	Amines
	1518	Urea
	1530	Aromatic amines
Juiciness	1162	C=O
	1452	Starch
	1772	Cellulose
Fatness	1162	C=O
	1488	CONHR, Amides, Aromatic amines
	1520	Urea
	1720	CO-Oil *
Fibrousness	1148	CH_2_ aromatic compounds
	1518	Urea
	1528	R-NH_2_
	1722	C-O-Oil *
	1736	SH- SH- *
	1928	Cellulose, Starch
	1956	Second overtone of CO_2_R
Chewiness	1218	-CH_2_
	1458	Starch
	1490	Amides, Urea, Aromatic amines, Starch, Cellulose
	1514	Protein
	1736	SH- SH- *
Gumminess	1488	CONR, Cellulose, Urea
	1504	Amines
	1526	Aromatic amines
	1736	SH- SH- *
	1746	SH- *
	1928	Cellulose, Starch
Heterogeneity	1510	Protein
	1534	R-N H_2_
	1922	Cellulose
Chewing Residue	1218	CH-CH_2_
	1514	Protein
	1530	Aromatic amines
	1618	=CH_2_

* Chemical structure and functional groups which have greater weight in the NIR models for predicting sensory attributes

**Table 4:** ANN architecture (training times and the number of neurons in the hidden layer) for each of the sensory parameters analyzed.

Sensory Attribute	The Best ANN Architecture (Higher R^2^ Value)	
	No. of Neurons in the Hidden Layer	No. of Training Times
Veined	6	500
Fat color	17	30
Color homogeneity	8	100
Color intensity	1	500
Exudate	6	500
White dots	3	500
Odor	10	500
Cured aroma	10	500
Pig aroma	27	30
Rancidity aroma	25	30
Atypical aroma	6	500
Flavor intensity	1	500
Fat flavor intensity	9	500
Cured flavor	6	500
Saltiness	7	500
Sweetness	23	30
Sourness	10	500
Rancidity	8	500
Aftertaste	27	30
Atypical flavor	3	100
Hardness	9	500
Juiciness	4	100
Fatness	7	500
Fibrousness	7	100
Chewiness	6	100
Gumminess	9	500
Heterogeneity	3	500
Chewing Residue	1	500

**Table 5:** The best ANN architecture and the mean square error of prediction (MSE) for each of the sensory parameters, obtained in the test set.

Sensory Attribute	No. of Neurons in the Hidden Layer	No. of Training Times	MSE
Veined	6	500	0.587
Fat color	17	30	0.088
Color homogeneity	8	100	0.110
Color intensity	1	500	0.081
Exudate	6	500	0.216
White dots	3	500	0.374
Odor	10	500	0.047
Cured aroma	10	500	0.031
Pig aroma	27	30	0.020
Rancidity aroma	25	30	0.025
Atypical aroma	6	500	0.019
Flavor intensity	1	500	0.039
Fat flavor intensity	9	500	0.216
Cured flavor	6	500	0.053
Saltiness	7	500	0.054
Sweetness	23	30	0.078
Sourness	10	500	0.029
Rancidity	8	500	0.044
Aftertaste	27	30	0.053
Atypical flavor	3	100	0.066
Hardness	9	500	0.204
Juiciness	4	100	0.077
Fatness	7	500	0.183
Fibrousness	7	100	0.205
Chewiness	6	100	0.242
Gumminess	9	500	0.207
Heterogeneity	3	500	0.086
Chewing Residue	1	500	0.107
